# Age- and Brain Region-Specific Changes of Glucose Metabolic Disorder, Learning, and Memory Dysfunction in Early Alzheimer’s Disease Assessed in APP/PS1 Transgenic Mice Using ^18^F-FDG-PET

**DOI:** 10.3390/ijms17101707

**Published:** 2016-10-18

**Authors:** Xue-Yuan Li, Wei-Wei Men, Hua Zhu, Jian-Feng Lei, Fu-Xing Zuo, Zhan-Jing Wang, Zhao-Hui Zhu, Xin-Jie Bao, Ren-Zhi Wang

**Affiliations:** 1 Department of Neurosurgery, Peking Union Medical College Hospital, Chinese Academy of Medical Sciences and Peking Union Medical College, Beijing 100730, China; lixueyuan-82@163.com (X.-Y.L.); zfx819_1986@163.com (F.-X.Z.); 2 Center for Magnetic Resonance Imaging, Peking University, Beijing 100871, China; wmen@pku.edu.cn; 3 Department of Pathology, Comparative Medical Center, Peking Union Medical College & Institute of Laboratory Animal Science, Chinese Academy of Medical Science, Beijing 100021, China; zhuhua0226@vip.sina.com (H.Z.); wangjingzhan@sina.com (Z.-J.W.); 4 Center for Medical Experiments and Testing, Capital Medical University, Beijing 100069, China; ccmu2013@sina.com; 5 Center for PET imaging, Peking Union Medical College Hospital, Chinese Academy of Medical Sciences and Peking Union Medical College, Beijing 100730, China; 13611093752@163.com

**Keywords:** hippocampus, glucose metabolism, ^18^F-FDG PET, cognitive dysfunction, APP/PS1 mice, Alzheimer’s disease

## Abstract

Alzheimer’s disease (AD) is a leading cause of dementia worldwide, associated with cognitive deficits and brain glucose metabolic alteration. However, the associations of glucose metabolic changes with cognitive dysfunction are less detailed. Here, we examined the brains of APP/presenilin 1 (PS1) transgenic (Tg) mice aged 2, 3.5, 5 and 8 months using ^18^F-labed fluorodeoxyglucose (^18^F-FDG) microPET to assess age- and brain region-specific changes of glucose metabolism. FDG uptake was calculated as a relative standardized uptake value (SUVr). Morris water maze (MWM) was used to evaluate learning and memory dysfunction. We showed a glucose utilization increase in multiple brain regions of Tg mice at 2 and 3.5 months but not at 5 and 8 months. Comparisons of SUVrs within brains showed higher glucose utilization than controls in the entorhinal cortex, hippocampus, and frontal cortex of Tg mice at 2 and 3.5 months but in the thalamus and striatum at 3.5, 5 and 8 months. By comparing SUVrs in the entorhinal cortex and hippocampus, Tg mice were distinguished from controls at 2 and 3.5 months. In MWM, Tg mice aged 2 months shared a similar performance to the controls (prodromal-AD). By contrast, Tg mice failed training tests at 3.5 months but failed all MWM tests at 5 and 8 months, suggestive of partial or complete cognitive deficits (symptomatic-AD). Correlation analyses showed that hippocampal SUVrs were significantly correlated with MWM parameters in the symptomatic-AD stage. These data suggest that glucose metabolic disorder occurs before onset of AD signs in APP/PS1 mice with the entorhinal cortex and hippocampus affected first, and that regional FDG uptake increase can be an early biomarker for AD. Furthermore, hippocampal FDG uptake is a possible indicator for progression of Alzheimer’s cognition after cognitive decline, at least in animals.

## 1. Introduction

Alzheime’s disease (AD) is a leading cause of dementia in adults that affects 46.0 million people worldwide [1]. Cognitive deficits are common in initial AD presentation, impairing daily performance and impacting quality of life, eventually ending in the death of the patient [2]. The pathophysiology of cognitive impairment caused by AD has not been elucidated, and it tends to be treated with routine clinical measures [3]. Cognitive deficits are often associated with brain metabolic alterations in AD [2,3], and studies using positron emission tomography (PET) have revealed brain glucose metabolic impairments in patients with AD [4]. However, a detailed analysis of the association between glucose metabolic dysfunction and specific cognitive impairments has not been conducted. Because learning and memory is first affected in AD, this study investigated glucose metabolic changes in specific brain regions of amyloid precursor protein (APP)/ presenilin 1 (PS1) transgenic (Tg) mice of varying ages using PET with learning and memory dysfunction resulting from early-stage AD.

PET provides a noninvasive measure to quantify brain glucose metabolism, and ^18^F-labed fluorodeoxyglucose (^18^F-FDG) is the most commonly used radiotracer analog of brain glucose [5,6]. Recent advances in scanner resolution have allowed quantitative measurement of regional glucose metabolic changes in mice brains using small animal PET (microPET) [3,5]. Because glucose is the major energy source for normal adult brains, the glucose metabolic rate can reflect neuronal function, synaptic density, and neuroinflammation. In early AD, neurons often exhibit a brief hyperglycolytic state in which glucose metabolism is enhanced to compensate for neuronal dysfunction [3]. Thereafter, a relatively longer period of metabolic depression follows, indicating irreversible neuronal damage [7,8]. Hypermetabolism before onset of clinical dementia could be an early biomarker for AD diagnosis [2,8]. However, the pattern of age- and brain region-specific changes in glucose metabolism and the best region for assessing glucose metabolism has not been determined.

The hippocampus is often the first-affected region in patients with AD [9]. The hippocampal damage is considered as a major contributor to the development of cognitive dysfunction in AD, including learning and memory impairments. A variety of studies have attempted to explore hippocampal function in AD-affected brains and explore its relationship with specific cognitive impairments, but most of them failed due to early limitation in imaging resolution [10,11]. However, an explicit study of the association between hippocampal glucose metabolism and hippocampus-dependent learning and memory impairment may facilitate future efforts towards the early diagnosis of AD and monitoring of disease progression.

The goal of this study was to evaluate glucose utilization in six brain regions (entorhinal cortex, hippocampus, frontal cortex, corpus callosum, striatum, thalamus) of four diverse age groups (2 months (pre-clinical), 3.5 months (sub-clinical), 5 months (early-clinical), 8 months (mid-clinical)) to assess age-specific changes in glucose metabolism in specifically designated brain regions in mice with an AD model and then explore their possible correlations with learning and memory dysfunctions; the purpose was to provide experimental cues for the management of AD patients using PET imaging.

## 2. Results

**Figure 1:**
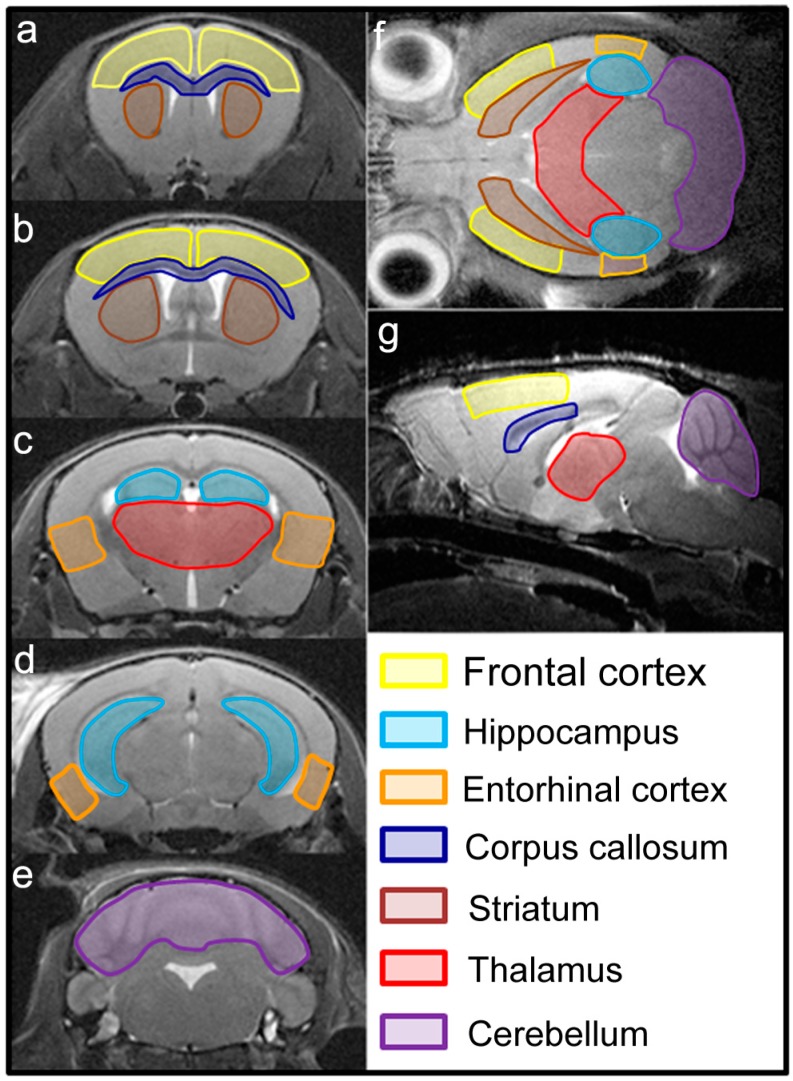
Seven regions of interest (ROI). The schematic boundaries of ROIs were drawn on the coronal (**a**–**e**), axial (**f**), and sagittal (**g**) T2-weighted images of a mouse brain according to the mouse brain atlas by Paxinos and Watson [12].

All tested mice completed the MWM, but four of them died during PET examination due to hyperanesthesia. The data of dead mice were included in the analysis of MWM results but excluded the correlation analysis due to the absence of corresponding PET data. All details of mice utilized in this study are summarized in Table 1. The regions of interest (ROIs) for PET data analysis are outlined in Figure 1.

### 2.1. Morris Water Maze (MWM) Results

**Figure 2:**
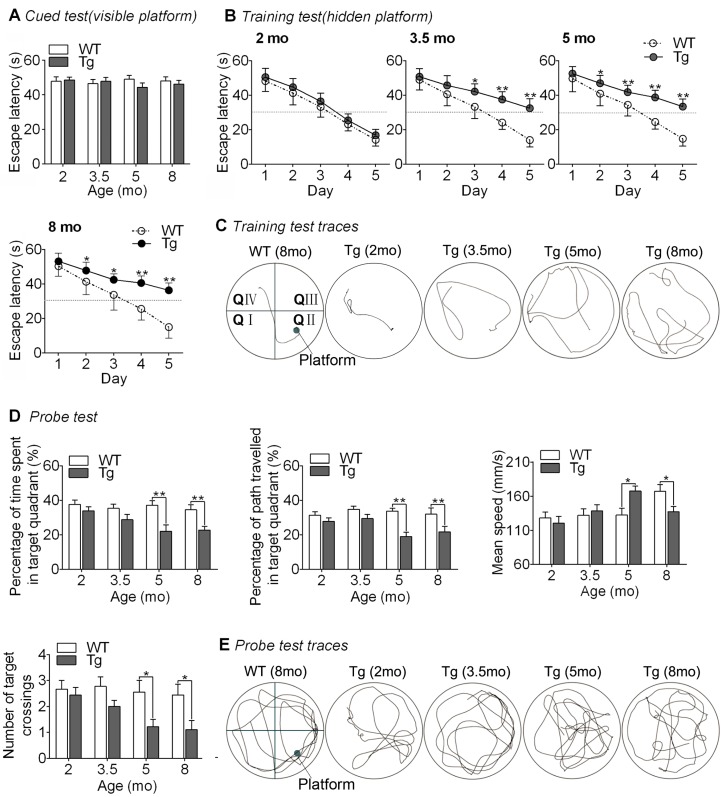
Morris water maze (MWM) results. (**A**) Comparisons of escape latencies in the cued test between Tg and wide-type (WT) mice aged 2, 3.5, 5 and 8 mo; (**B**) Escape latencies in 5 days in the training test and (**C**) typical training traces (the fifth day) for mice of each age. (* *p* < 0.05; ** *p* < 0.01; vs. aged-matched WT mice); (**D**) Comparisons of the percentage of time spent in the target quadrant, percentage of path travelled in the target quadrant, mean speed, as well as number of target crossings in the probe test between Tg and WT mice aged 2, 3.5, 5 and 8 mo; and (**E**) typical probe traces. (* *p* < 0.05; ** *p* < 0.01; vs. aged-matched WT mice). mo: month-old.

The MWM test was conducted to evaluate the spatial learning and memory of mice, and the results are summarized in Figure 2. The cued test was used to assess the visual acuity and motion behavior in water. All tested mice escaped on platform in at least one of the three trials and there was no notable difference in escape latency between Tg and wide-type (WT) mice at any age (2-way ANOVA, *F*_1,67_ = 0.194, *p* = 0.94) (Figure 2A), suggesting no notable age-related, visual and motion deficits in tested mice.

The training tests were used to assess spatial learning. Tg mice aged 2 mo (month-old) appeared to take more time to find the hidden platform compared with WT controls; however, no significant difference was found in escape latencies between the two groups (2-way repeated measures ANOVA, *F*_1,16_ = 1.92, *p* = 0.19) (Figure 2B). By contrast, Tg mice of 3.5 mo showed significantly longer escape latencies than age-matched WT controls (2-way repeated measures ANOVA, *F*_1,16_ = 21.1, *p* < 0.001) at the third day of training, and the latency difference tended to be more notable afterwards (Tukey’s Test, all *p* < 0.05) (Figure 2B), suggesting slower learning speed. Likewise, Tg mice aged 5 and 8 mo took more time to escape the pool than age-matched controls (2-way repeated measures ANOVA, 5 mo: *F*_1,16_ = 22.9, *p* < 0.001, and 8 mo: *F*_1,16_ = 27.8, *p* < 0.001), with latency differences appearing significant at the second training day (Tukey’s Test, all *p* < 0.05) (Figure 2B), suggesting learning deficits progressing with age in Tg mice. Figure 2C shows typical swimming traces at the fifth day of training for Tg and WT mice.

The probe test was used to assess memory retention. In the test, Tg mice aged 2 mo showed no notable discrimination from age-matched controls in any of the four tested parameters (2-way ANOVA Tukey’s Test, all *p* > 0.05) (Figure 2D), confirming intact spatial memory. However, in Tg mice of 3.5 mo, the percentage of time spent and path travelled in the target quadrant was reduced relative to the controls, but no significant differences were observed for any of the four tested parameters, including the swimming speed and number of target crossings (2-way ANOVA Tukey’s Test, all *p* > 0.05) (Figure 2D), suggesting affected but efficient spatial memory. At ages 5 and 8 mo, Tg mice showed significantly lower percentages of time and path in the target quadrant and significantly less platform crossings than age-matched controls (2-way ANOVA Tukey’s Test, all *p* < 0.05), indicating complete learning and memory deficits in Tg mice of two age groups (Figure 2D). However, compared with the controls, the swimming speed was notably increased in Tg mice at 5 mo but significantly reduced at 8 mo (2-way ANOVA Tukey’s Test, all *p* < 0.05), suggesting enabled neurological function to activate movements in Tg mice at 8 mo. Typical probe test traces for Tg and WT mice are shown in Figure 2E.

All results indicate that Tg mice showed intact cognition at age 2 mo, learning deficits at 3.5 mo, learning and memory deficits at 5 mo, and further learning and memory impairments at 8 mo. It seems that Tg mice of 2 mo mimicked the pre-clinical stage of AD (prodromal-AD), whereas Tg mice of 3.5, 5, and 8 mo exhibited sub-, early- and mid-clinical signs of AD (symptomatic-AD).

### 2.2. Age- and Region-Specific Changes in Brain Glucose Metabolism

**Figure 3:**
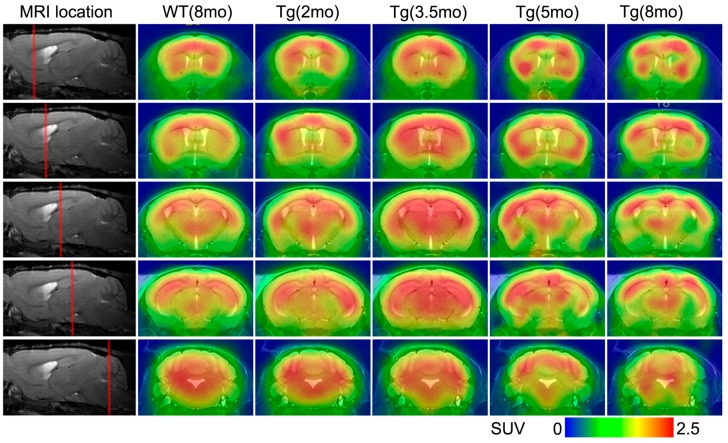
Results of PET/MRI fusion. Typical coronal ^18^F-FDG-PET images from WT (8 mo) and Tg mice (2, 3.5, 5 and 8 mo), projected on standard T2-weighed MR images from a WT mouse. mo: month-old.

^18^F-FDG-PET was used to reveal brain glucose metabolic changes in Tg and WT mice at ages 2, 3.5, 5 and 8 mo; typical PET images are shown in Figure 3. Compared with WT controls, Tg mice showed glucose utilization increase in several brain regions, including the entorhinal cortex, hippocampus and frontal cortex, at age 2 mo; this increase was also present, with more brain regions affected, at age 3.5 mo. In Tg mice of 5 and 8 mo, increased glucose metabolism appeared to decline in the entorhinal cortex and hippocampus but tended to intensify in the thalamus and striatum, with asymmetrical glucose disorders distributed in discrete regions; the asymmetry was more pronounced at age 8 mo. By contrast, T2-weighted MR images showed no visible structural changes in any brain region of Tg mice at any age.

**Figure 4:**
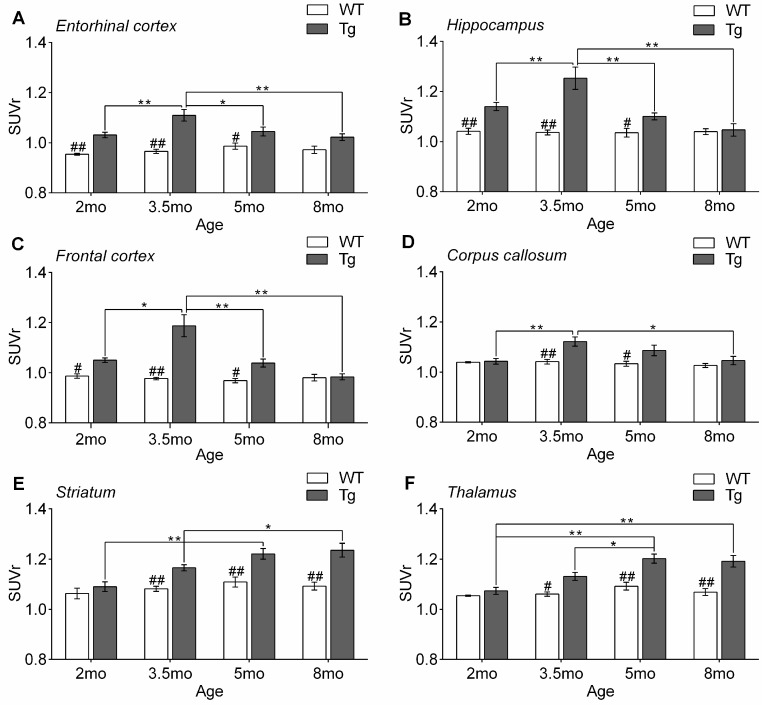
Quantitative analysis of PET data. ROI-based SUVr (relative standardized uptake value) group comparisons for Tg and WT mice aged 2, 3.5, 5 and 8 mo. Graphs highlight age-specific changes in SUVr values for the entorhinal cortex (**A**); hippocampus (**B**); frontal cortex (**C**); corpus callosum (**D**); striatum (**E**); and thalamus (**F**). (* *p* < 0.05, ** *p* < 0.01, vs. Tg mice of other age groups; # *p* < 0.05, ## *p* < 0.01 vs. Tg mice of the same age group). mo: month-old.

A quantitative analysis of PET images was conducted according to ROIs outlined in Figure 1 (Figure 4). A 2-way ANOVA showed significant group differences in SUVr (relative standardized uptake value) between Tg and WT mice in all tested brain regions (entorhinal cortex: *F*_1,63_ = 54.4, *p* < 0.001; hippocampus: *F*_1,63_ = 34.4, *p* < 0.001; frontal cortex: *F*_1,63_ = 30.1, *p* < 0.001; corpus callosum: *F*_1,63_ = 14.6, *p* < 0.001; striatum: *F*_1,63_ = 41.8, *p* < 0.001, and thalamus: *F*_1,63_ = 46.9, *p* < 0.001). Compared with that of age-matched WT mice, SUVr values in the entorhinal cortex, hippocampus and frontal cortex of Tg mice were significantly elevated at age 2 mo, further increased at age 3.5 mo, but declined notably at age 5 mo, and reduced to basal levels at age 8 mo (2-way ANOVA Tukey’s Test, all *p* > 0.05) (Figure 4A–C). By contrast, SUVr values in the striatum and thalamus of Tg mice increased progressively with age after reaching significance at age 3.5 mo (2-way ANOVA Tukey’s Test, all *p* < 0.05) (Figure 4E,F). When ROI-specific SUVr values were compared across Tg mice of diverse ages, Tg mice of 3.5 mo showed the highest SUVr in the entorhinal cortex, hippocampus and frontal lobe (Figure 4A–C), whereas Tg mice of 8 mo showed the highest SUVr in the striatum and thalamus, compared with Tg mice of other ages (Figure 4E,F).

### 2.3. Correlation Analysis between Hippocampal SUVr (Relative Standardized Uptake Value) and MWM Parameters

**Figure 5:**
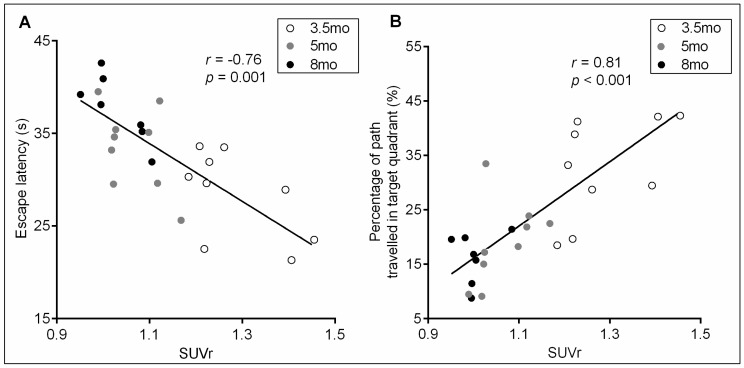
Correlations of hippocampal glucose utilization with MWM performance in mice at ages 3.5, 5 and 8 mo. Hippocampal SUVr values of individual animals were correlated with the escape latency (**A**) and percentage of path travelled in the target quadrant (**B**). A linear regression model was used for the correlation analysis. The regression curve line, correlation coefficients (*r*), and significance values are shown in the plots. Statistical significance was set at *p* < 0.05. mo: month-old.

A correlation analysis between hippocampal SUVr values and MWM parameters were performed in Tg mice to test whether glucose disorder in the hippocampus can indicate cognitive dysfunction. Because escape latency and percentage of path in the target quadrant are most commonly used in the MWM, they were used for the analysis, wherein latency data were collected from the fifth training day. Because Tg mice of 2 mo showed normal performance in the MWM, only the data from Tg mice of 3.5 to 8 mo were included in the analysis. Pearson correlation tests showed that there was a close and highly significant relationship between hippocampal SUVr and escape latency (*r* = −0.78, *p* = 0.001) and percentage of path in the target area (*r* = 0.81, *p* < 0.001) (Figure 5), suggesting a notable correlation between changes in hippocampal glucose and changes in Alzheimer’s cognition after the onset of symptoms. To determine the effect of age on the correlation, a multiple linear correlation analysis with age group as a variable was further conducted. Results showed that age has non-significant correlations with escape latency (*r* = 0.177, *p* = 0.440) and percentage of path in the target quadrant (*r* = −0.154, *p* = 0.472), suggesting that Tg mice of the three age groups share uniform correlations between hippocampal SUVr and MWM performance.

### 2.4. Histological Confirmation of Alzheimer’s Pathology

**Figure 6:**
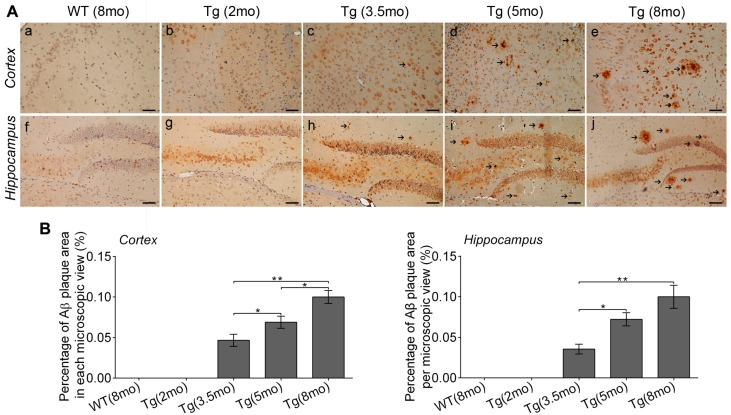
Immunohistochemical results. (**A**) Representative images of Aβ and plaques in the entorhinal cortex and hippocampus (dental gyrus) of WT and Tg mice aged 2, 3.5, 5 and 8 mo, visualized by staining Aβ_1–16_ peptides. Arrows indicate the presence of Aβ plaques. Scale bar: 50 μm; (**B**) Percentage of Aβ plaque area in each microscopic view (at 200×) in the entorhinal cortex and hippocampus in Tg and WT mice aged 2, 3.5, 5 and 8 mo. (* *p* < 0.05, ** *p* < 0.01, vs. Tg mice of other age groups). mo: month-old.

The anti-Aβ1-16 antibody was used to stain Aβ peptides to confirm Alzheimer’s pathology in Tg mice; typical images are shown in Figure 6. Although Tg mice aged 2 mo already showed visible Aβ staining in the entorhinal cortex and hippocampus, there were no signs of Aβ plaques in these regions, suggesting the overproduction of Aβ peptides (Figure 6A). Spot-like Aβ staining appeared in the entorhinal cortex and hippocampus of Tg mice at age 3.5 mo, and the number of Aβ plaques (Figure 6A, arrow) and percentage of plaque area in each microscopic view was markedly increased in Tg mice aged 5 and 8 mo (one-way ANOVA Tukey’s Test, all *p* < 0.05) (Figure 6B). However, sporadic staining of Aβ plaques in the corpus callosum was only observed in Tg mice at age 8 mo. No Aβ-stained deposits were detected in WT mice of any age (Figure 6A).

## 3. Discussion

To provide basic evidence regarding PET scanning in patients with probable AD, we used microPET to study age- and brain region-specific changes of early glucose disorder and learning and memory dysfunction in APP/PS1 Tg mice. We found a significant increase in brain glucose metabolism before cognitive decline in Tg mice, with the largest increases observed in the entorhinal cortex, hippocampus and frontal cortex. After the onset of cognitive deficits, glucose utilization tended to decline in the entorhinal cortex and hippocampus of Tg mice but to increase in the striatum and thalamus. By comparing SUVrs in the entorhinal cortex and hippocampus, Tg mice were distinguished from controls before cognitive decline. In addition, hippocampal glucose metabolic changes were significantly associated with learning and memory deficits after the onset of AD symptoms. These results suggest that ^18^F-FDG-PET can reveal glucose metabolic disorders in early AD and indicate disease progression after cognitive decline. This is the first study, to our knowledge, that details the changes of glucose metabolism in early AD and explores their relationship with the development of specific cognitive deficits.

Growing evidence has shown that patients with AD often experience a lengthy (>10 years) period of disease progression before cognitive decline [13]. This period often involves several pathophysiological variables and complex interactions, including glucose metabolic disorders. Elucidation of the disorders can facilitate early AD diagnosis. However, most patients already have late-stage AD at diagnosis because of the prior absence of featured clinical signs; thus, it is impractical to explore early AD-related pathophysiological changes in patients. Model animals facilitate the study of pathological changes in early AD, wherein APP/PS1 Tg mice have been widely used to investigate early pathogenic changes similar to human AD [14]. In this study, Tg mice showed complete AD-like cognitive deficits at ages 5 and 8 mo and a slower learning speed at age 3.5 mo but not age 2 mo. However, staining of Aβ peptides was already present in Tg mice at age 2 mo, Aβ plaques appeared at age 3.5 mo, and the plaque area increased with age thereafter. Thus, based on our and previous findings [13,15], Tg mice of four ages used in this study mimicked the pre-, sub-, early- and mid-clinical stage of AD.

Using microPET, we showed noticeable increases in glucose metabolism in several brain regions of Tg mice aged 2 and 3.5 mo compared with aged-matched controls; the increased glucose metabolism declined at 5 mo and returned to basic levels at 8 mo. With an image fusion technique [16], we coregistered PET images with MR images and calculated SUVr values in specific brain regions. Results showed that metabolic increase was major in the entorhinal cortex and hippocampus of Tg mice aged 2 mo (before cognitive decline), which was notably different from that of the controls. In these regions, the increased SUVr values were more prominent at 3.5 mo, but significantly reduced to the same levels as the control in Tg mice of 5 and 8 mo (after cognitive decline). These findings suggest that PET detected early abnormalities in glucose metabolism in mice with AD and that FDG uptake can be an early biomarker for AD diagnosis. By contrast, SUVr in the striatum and thalamus of Tg mice was significantly elevated at age 3.5 mo and progressively elevated thereafter. These findings are consistent with previous functional MRI studies of brain regions vulnerable to AD [17,18,19] and imply that the entorhinal cortex and hippocampus can be target sites for AD detection via glucose metabolism.

There are several causes explaining the age- and brain region-specific changes of glucose metabolic disorder observed in early AD. The initial brain hyperglycolysis could be associated with compensatory activation of existing neurons for lost ones in AD-affected brain regions [20]. Because glucose is the brain’s major energy source, the compensation would metabolize more glucose to maintain neurological function [21]. In this phase, the Aβ overexpression and accidental seizure activity also require more glucose utilization for energy, contributing to the hyperglycolysis. The subsequent glucose utilization reduction may be caused by Aβ overload and excessive neuronal loss [22]. While Aβ overload adversely affects glucose utilization, neuronal loss leads to the immediate reduction of glucose metabolism. When existing neurons cannot take over lost ones, glucose metabolism begins to decline. Moreover, innate immune-mediated neuroinflammation also occurs in company with Aβ accumulation in AD, which often leads to abundant inflammatory cells, including glial activation that expresses high levels of glucose transporters and thus increases brain glycolytic activity [23,24,25]. Our study revealed the entorhinal cortex and hippocampus as regions affected first with glucose disorders, perhaps because these regions are highly dependent on the insulin signaling pathway and are initially subject to Aβ accumulation [26,27]. While accumulated Aβ causes glucose metabolic disorder by disturbing the insulin signaling pathway, altered glucose metabolism can conversely promote Aβ accumulation. Our histological examination showed that glucose utilization increased with emerging Aβ deposition but attenuated with growing Aβ plaques, supporting the contribution of Aβ deposition to glucose disorders. However, no direct correlational analysis was conducted between the two factors in this study, and future researches are required to evaluate the relationship.

However, the early glucose metabolic increase, as we observed in Tg mice, is inconsistent with many clinical reports. For example, Kantarci et al. examined patients with early AD using FDG-PET, finding reduction of glucose metabolism in several brain regions including the temporal and parietal lobes [28]. Also, Herholz et al. reported a decline of FDG uptake in the temporoparietal and prefrontal cortex of patients with probable AD [29]. The inconsistence, on one aspect, could be related with diverse histological constitution in the brain between mice and humans. Specifically, the volume ratio of grey matter in the whole brain is notably higher in mice than in humans [30], and thus mice brains have more neurons to consume in the degenerative process. On another aspect, due to a long asymptomatic period, patients consenting to PET examination may be already at late-stage AD. This is supported by studies showing hyperglycolysis in the cortex and medial temporal lobe of patients who are suspected to have AD [31,32]. There are also studies reporting that glucose metabolism began to decline after cognitive deficits appeared in AD patients, suggesting that there may be ever hyperglycolysis in the brain. It is possible that APP/PS1 mice represent an early stage of AD or the metabolic data from AD patients are collected at the late stage of the disease.

Consistent with our findings, two other PET studies using APP/PS1 mice also showed an initial glucose metabolic increase and subsequent metabolic reduction in the brain [8,33]. These results suggest that glucose metabolic disorders occur in the brain of Tg mice before cognitive decline and that disordered glucose metabolism can differentiate AD from the controls; this is congruent between animals and humans and provides the basis for studying metabolic changes occurring in human AD using animals. However, the difference in glucose metabolism between animals and humans should be considered when extrapolating the results obtained from mice.

Besides detailing changes in regional glucose metabolism, we also explored the association of hippocampal glucose metabolic changes with learning and memory dysfunctions in Tg mice with cognitive deficits. The hippocampus was selected because it is closely related with learning and memory formation and its damage is thought to vitally involve the AD process. Also, studies have shown that the hippocampus is an initial site of Aβ accumulation and neuronal damage [19,34], offering a pathologic basis for AD-related cognitive deficits. Once hippocampal damage reaches a certain level, resulting learning and memory deficits can be evaluated by the MWM in mice. From our correlation analysis, it seems that hippocampal SUVrs had significantly negative correlation with escape latency but positive relationship with percentage of path in the target quadrant in Tg mice with symptomatic AD. Thus, because increased escape latency and reduced percentage of path in the target quadrant both indicate cognitive deficits, lower SUVrs should point to serious cognitive impairment while higher SUVrs indicate mild impairment in APP/PS1 mice, suggesting that hippocampal FDG uptake can be an indicator of the progression in Alzheimer’s cognition, at least in animals. Such a finding is consistent with the reports by Herhoiz et al. [35], who showed that a calibrated FDG-PET score can serve as a biomarker for progression in AD clinically. However, it must be noted that the baseline of FDG uptake in this study was from Tg mice with sub-clinical AD in which glucose metabolism was notably enhanced compared with the controls. Thus, it is required to learn the base FDG uptake when using PET to monitor progression in AD mice.

This study has some limitations, notably the lack of within-subjects design. The mice of different age groups were from different cohorts but tested at the same time. Although the animal selection and test timing were strictly controlled, there were always within-animal changes in glucose metabolism, which need to be weighed into this study’s findings. For another, the model animals used in this study exhibited typical Aβ depositions similar to human AD but no neurofibrillary tangles (NFTs), another pathological feature of AD. However, NFTs are often present in the mid- to late-stage of AD, whereas this study focuses on metabolic changes in early AD; thus the findings have certain universal significance. Next, the spillover and partial volume effects inherent in PET imaging challenge the measurement of radioactivity in small ROIs; such limitations of microPET limit the ROI definition and radioactivity measurement. Thus, PET images were fused with T2-weighted images to accurately locate ROIs and only larger-sized ROIs were included to minimize volume effects. Moreover, this study employed static PET to correlate SUVr values with activated MWM parameters, but the quantification may be better by using dynamic PET to estimate the glucose metabolic rate from FDG (MRFDG). However, SUVs have been shown to correlate well with percentage changes in MRFDG (*r* = 0.84; [36]); thus, static PET reflects brain activation during the MWM. Thus, this study’s findings represent a conservative revelation of brain abnormalities occurring in early AD.

## 4. Materials and Methods

### 4.1. Animals

All animal experiments were approved by the Institutional Animal Care and Use Committee of the Institute of Laboratory Animal Science of Peking Union Medical College (ILAS-PL-2014-003, Date of approval: 24 October 2014). Animals were provided by the Institute of Experimental Animals of the Chinese Academy of Medical Science and cared for according to the guidelines published in the National Institutes of Health Guide for Care and Use of Laboratory Animals.

APP/PS1 Tg mice (C57BL/6J) used in this study were produced by co-injecting APPswe and PS1ΔE9 vectors as described [37]. These mice overexpress the Swedish (K594M/N595L) mutation of human APP together with PS1 deleted in exon 9 driven by the mouse prion protein promoter, confirmed by PCR genotyping of mouse tail tissue [37,38]. Only female mice were utilized because the female APP/PS1 mice develop cognitive deficits faster than the male mice. Thirty-six Tg mice and 36 age-matched WT littermates were tested at ages 2, 3.5, 5 and 8 mo (*n* = 9 Tg and 9 WT in each age group).

### 4.2. MWM

The WMM test, which has been widely used in studies of AD models in rodents [16,39], was used to assess the learning and memory of mice in this study. The maze was located in a white circular pool (100 cm diameter and 50 cm height) divided into four equal quadrants. The pool was filled with water at 22 ± 1 °C and made opaque with nonfat milk. At the start, a cued test, including three trials of 60 s, in which there was a visible escape platform positioned in the center of quadrant IV was conducted to assess age-related visual deficits and motion to escape from water. A flag was positioned on the platform, which the mice could use to navigate the maze. Subsequently, the training tests were performed in succession. In the training tests, the platform was placed in the center of quadrant II, submerged 1 cm below the water surface, with a cue flag positioned on the wall surrounding the pool for navigation. Each mouse was given three trials from different starting points, each lasting 60 s, for 5 consecutive days, in which the mice were allowed to swim freely to the platform. Finally, at 24 h after the last set of training trials, a probe test was started, during which the platform was removed from the pool and the mouse dropped at a position opposite quadrant II. After each trial, the mice were kept in a plastic holding cage placed on an electric heater. The swimming of mice was recorded with a video connected to a computer-driven movement tracing system (Ethovision, Noldus Information Technology, Wageningen, The Netherlands). The escape latency (time to reach the platform) was analyzed and recorded as averages for the cued and training tests. The percentage of time spent in the target quadrant, the percentage of distance travelled in the target quadrant, the swimming speed, and the crossings of the target platform were calculated for the probe test.

### 4.3. MicroPET Imaging

The mice were subjected to FDG-PET scans using a microPET scanner (Inveon, Siemens Medical Solutions, Knoxville, TN, USA). All mice fasted ≥6 h before PET scans to achieve a stable plasma glucose level. After an intravenous injection of FDG (diluted in saline, 111 MBq/kg), each animal was kept in an individual cage in a quiet, lightly dim and warm room. Forty minutes later, the mice were anesthetized via inhalation of 5% isoflurane/95% O_2_ mixture, placed in the prone positon, and maintained under anesthesia with 2% isoflurane/98% O_2_ mixture. The body temperature of animals was maintained at 37 °C using a circulating water heating pad. Then, a static PET scan was performed continuously for 23 min (spatial resolution: 1.35 mm; field of view (FOV): 12.7 cm), followed by a transmission scan from a rotating ^57^Co point source for attenuation correction. The PET images were reconstructed using a two-dimensional filtered back-projection algorithm, resulting in 0.78 mm × 0.78 mm × 0.80 mm voxel size. The images were reconstructed in axial, coronal and sagittal views on the Inveon Research Workplace 3.0 software package (Siemens Medical Solutions). After PET scanning, each mouse underwent a brain MRI using a 7.0-T MR scanner (V.70/16; PharmaScan, Bruker Biospin, Rhein-stetten, Germany) to acquire high-resolution T2-weighted MR images (TE = 24 ms; TR = 4000 ms; FOV = 20 mm × 20 mm; matrix = 256 × 256). The acquired images were used for observing brain structural changes associated with AD and then fusing with PET images. After spatial transformation, T2-weighted images were coregistrated with the corresponding PET images using SPM12 software (Wellcome Department of Cognitive Neurology, Institute of Neurology, London, UK), as described previously [16]. The resulting PET/MR fusion images were resliced with trilinear interpolation to produce 0.4-mm-thick image slices for analysis.

### 4.4. PET Data Analysis

FDG-PET data were analyzed using the ImageJ software package (National Institutes of Health, Bethesda, MD, USA), as described previously [16]. ROIs were manually drawn on brain MRI slices according to the mouse brain atlas by Pixinos and Wastson [12]. Boundaries of ROIs, including the frontal cortex, hippocampus, entorhinal cortex, corpus callosum, striatum, thalamus and cerebellum, were drawn at optimal coronal layers and confirmed on axial and sagittal MRI slices (Figure 1). The ROIs were then transferred to identical sites on the PET/MRI fusion and PET images of the same mouse. The quantitative parameters of each ROI were calculated as average values from three consecutive slices. Bilateral ROIs from the same mouse were analyzed in combination. For quality control, the ROIs were drawn by a blinded investigator who was instructed to make them sufficiently large.

Brain ^18^F-FDG uptake was calculated as the SUV, corrected for injection time and divided by the injected dose and animal weight. The SUV data within ROIs were collected for each mouse. To keep consistent across animals, relative SUV (SUVr) was calculated for each ROI with the cerebellum as referencing tissue because the cerebellum is minimally affected in glucose metabolism [40]. SUVr values from different ROIs were compared within brains and within age groups to evaluate the age- and brain region-specific changes in glucose metabolism. Hippocampal SUVr values were evaluated for their potential relationship with MWM parameters.

### 4.5. Immunohistochemical Analysis

After MR scanning, for histological examination, all mice were subject to cardiac perfusion with 4% paraformaldehyde while deeply anesthetized, and then the brains were removed and post fixed for at least 24 h. The fixed brains were then dehydrated by gradient ethanol, embedded in paraffin and cut into 6-μm coronal sections. Brain sections were stained with anti-Aβ_1–16_ antibody at 1/500 strength (6E10; Covance/Signet Laboratories, Dedham, MA, USA), followed by secondary antibody (HRP-labeled anti-rabbit/mouse IgG) and DAB (ZSJQ-BIO, Beijing, China), to identify Aβ deposition in plaques. Counterstaining was performed on representative sections with hematoxylin, and then the sections were subject to image analysis (Leica TCS SP2, Leica, Wetzlar, Germany) and analyzed using the Image Pro Plus 6.0 software package (Media Cybernetics Inc., Rockville, MD, USA) to calculate the percentage of Aβ plaque area in each microscopic view at 200× magnification in the entorhinal cortex and hippocampus.

### 4.6. Statistical Analysis

All results were expressed as means ± SEM. Statistical analyses were performed using GraphPad Prism 6.0 (GraphPad Software, Inc., La Jolla, CA, USA). One or two-way ANOVA were used for multiple group analysis, followed by Tukey’s Test (α = 0.05) to assess the between-group difference. Linear regression analysis was used to predict the association between hippocampal SUVr values and MWM parameters in Tg mice. Differences with *p*-values of <0.05 were considered statistically significant.

## 5. Conclusions

In summary, this study used ^18^F-FDG microPET in combination with the MWM test to assess age- and brain region-specific changes of glucose metabolic disorder and explore their associations with learning and memory dysfunction in APP/PS1 Tg mice. Results showed that glucose metabolism was significantly increased in multiple brain regions of Tg mice with early AD and that the entorhinal cortex, hippocampus and frontal cortex were the first sites affected with glucose metabolic disorders. Increased glucose utilization in the entorhinal cortex and hippocampus can differentiate Tg mice from WT controls before onset of cognitive deficits. Hippocampal SUVr changes were significantly correlated with MWM parameters after cognitive deficits. These findings suggest regional FDG uptake increase can be used as an early biomarker for AD diagnosis in Tg mice. In addition, hippocampal FDG uptake can be an indicator for AD progression after cognitive deficits, at least in animals. Further studies are required to validate whether or to what extent the results obtained in this study can be extrapolated to patients in clinic.

## Figures and Tables

**Table 1:** Demographics and overview of mice used in the study.

Mouse Type	Sex	Age (mo)	*n*	Weight (g)	PET Scan Completion (*n*)	Drop out Due to Death (*n*)
APP/PS1	F	2	9	18.3 ± 1.9	8	1
	F	3.5	9	23.4 ± 2.2	9	0
	F	5	9	27.4 ± 3.2	9	0
	F	8	9	29.2 ± 3.9	7	2
WT	F	2	9	18.8 ± 1.4	9	0
	F	3.5	9	24.5 ± 1.8	9	0
	F	5	9	28.2 ± 2.6	9	0
	F	8	9	30.6 ± 3.5	8	1

APP: amyloid precursor protein; F: female; mo: month-old; *n*: number; PET: positron emission tomography; PS1: presenilin 1; WT: wide-type.

## Abbreviations

AD	Alzheimer’s disease
FDG	fluorodeoxyglucose
APP	precursor protein
PS1	presenilin 1
Tg	transgenic
WT	wide-type
mo	month-old
MWM	morris water maze
SUV	standardized uptake value
ANOVA	analysis of variance

## References

[1] Li X.Y., Bao X.J., Wang R.Z. (2015). Potential of neural stem cell-based therapies for Alzheimer’s disease. J. Neurosci. Res..

[2] Herholz K. (2010). Cerebral glucose metabolism in preclinical and prodromal Alzheimer’s disease. Expert Rev. Neurother..

[3] Shokouhi S., Claassen D., Kang H., Ding Z., Rogers B., Mishra A., Riddle W.R., Alzheimer’s Disease Neuroimaging Initiative (2013). Longitudinal progression of cognitive decline correlates with changes in the spatial pattern of brain 18F-FDG PET. J. Nucl. Med..

[4] Zippo A.G., Castiglioni I. (2015). Integration of 18FDG-PET metabolic and functional connectomes in the early diagnosis and prognosis of the Alzheimer’s disease. Curr. Alzheimer Res..

[5] Xi W., Su D., Nie B., Yu Y., Shan B., Chen Q., Tian M., Zhang H. (2013). 18F-FDG PET study reveals brain functional changes during attention in rats. J. Nucl. Med..

[6] Palumbo B., Buresta T., Nuvoli S., Spanu A., Schillaci O., Fravolini M.L., Palumbo I. (2014). Spect and pet serve as molecular imaging techniques and in vivo biomarkers for brain metastases. Int. J. Mol. Sci..

[7] Verclytte S., Lopes R., Lenfant P., Rollin A., Semah F., Leclerc X., Pasquier F., Delmaire C. (2015). Cerebral hypoperfusion and hypometabolism detected by arterial spin labeling mri and FDG-PET in early-onset Alzheimer’s disease. J. Neuroimaging.

[8] Poisnel G., Herard A.S., El Tayara N.E., Bourrin E., Volk A., Kober F., Delatour B., Delzescaux T., Debeir T., Rooney T. (2012). Increased regional cerebral glucose uptake in an APP/PS1 model of Alzheimer’s disease. Neurobiol. Aging.

[9] Fiorenza N.G., Rosa J., Izquierdo I., Myskiw J.C. (2012). Modulation of the extinction of two different fear-motivated tasks in three distinct brain areas. Behav. Brain Res..

[10] Wang Z. (2014). Characterizing early Alzheimer’s disease and disease progression using hippocampal volume and arterial spin labeling perfusion MRI. J. Alzheimer’s Dis..

[11] Velez-Pardo C., Arellano J.I., Cardona-Gomez P., del Rio M.J., Lopera F., de Felipe J. (2004). CA1 hippocampal neuronal loss in familial Alzheimer’s disease presenilin-1 E280A mutation is related to epilepsy. Epilepsia.

[12] Konsman J.P., Franklin K.B.J., Paxinos G. (2001). The Mouse Brain in Stereotaxic Coordinates, Second Section.

[13] Hampel H., Prvulovic D., Teipel S., Jessen F., Luckhaus C., Frolich L., Riepe M.W., Dodel R., Leyhe T., Bertram L. (2011). The future of Alzheimer’s disease: The next 10 years. Prog. Neurobiol..

[14] Li X., Bao X., Wang R. (2016). Experimental models of Alzheimer’s disease for deciphering the pathogenesis and therapeutic screening (Review). Int. J. Mol. Med..

[15] Webster S.J., Bachstetter A.D., Nelson P.T., Schmitt F.A., van Eldik L.J. (2014). Using mice to model Alzheimer’s dementia: An overview of the clinical disease and the preclinical behavioral changes in 10 mouse models. Front. Genet..

[16] Li J., Gu L., Feng D.F., Ding F., Zhu G., Rong J. (2012). Exploring temporospatial changes in glucose metabolic disorder, learning, and memory dysfunction in a rat model of diffuse axonal injury. J. Neurotrauma.

[17] Platt B., Welch A., Riedel G. (2011). FDG-PET imaging, EEG and sleep phenotypes as translational biomarkers for research in Alzheimer’s disease. Biochem. Soc. Trans..

[18] Landau S.M., Lu M., Joshi A.D., Pontecorvo M., Mintun M.A., Trojanowski J.Q., Shaw L.M., Jagust W.J., Alzheimer’s Disease Neuroimaging Initiative (2013). Comparing positron emission tomography imaging and cerebrospinal fluid measurements of beta-amyloid. Ann. Neurol..

[19] Murray M.E., Przybelski S.A., Lesnick T.G., Liesinger A.M., Spychalla A., Zhang B., Gunter J.L., Parisi J.E., Boeve B.F., Knopman D.S. (2014). Early Alzheimer’s disease neuropathology detected by proton MR spectroscopy. J. Neurosci..

[20] Nicholson R.M., Kusne Y., Nowak L.A., LaFerla F.M., Reiman E.M., Valla J. (2010). Regional cerebral glucose uptake in the 3XTG model of Alzheimer’s disease highlights common regional vulnerability across AD mouse models. Brain Res..

[21] Sancheti H., Patil I., Kanamori K., Diaz Brinton R., Zhang W., Lin A.L., Cadenas E. (2014). Hypermetabolic state in the 7-month-old triple transgenic mouse model of Alzheimer’s disease and the effect of lipoic acid: A 13C-NMR study. J. Cereb. Blood Flow Metab..

[22] Sun Y., Rong X., Lu W., Peng Y., Li J., Xu S., Wang L., Wang X. (2015). Translational study of Alzheimer’s disease (AD) biomarkers from brain tissues in AβPP/PS1 mice and serum of AD patients. J. Alzheimer’s Dis..

[23] Jayasena T., Poljak A., Braidy N., Smythe G., Raftery M., Hill M., Brodaty H., Trollor J., Kochan N., Sachdev P. (2015). Upregulation of glycolytic enzymes, mitochondrial dysfunction and increased cytotoxicity in glial cells treated with Alzheimer’s disease plasma. PLoS ONE.

[24] Aso E., Lomoio S., Lopez-Gonzalez I., Joda L., Carmona M., Fernandez-Yague N., Moreno J., Juves S., Pujol A., Pamplona R. (2012). Amyloid generation and dysfunctional immunoproteasome activation with disease progression in animal model of familial Alzheimer’s disease. Brain Pathol..

[25] Rojas S., Herance J.R., Gispert J.D., Abad S., Torrent E., Jimenez X., Pareto D., Perpina U., Sarroca S., Rodriguez E. (2013). In vivo evaluation of amyloid deposition and brain glucose metabolism of 5XFAD mice using positron emission tomography. Neurobiol. Aging.

[26] Van den Hove D.L., Kenis G., Brass A., Opstelten R., Rutten B.P., Bruschettini M., Blanco C.E., Lesch K.P., Steinbusch H.W., Prickaerts J. (2013). Vulnerability versus resilience to prenatal stress in male and female rats; implications from gene expression profiles in the hippocampus and frontal cortex. Eur. Neuropsychopharmacol..

[27] Katafuchi T., Ifuku M., Mawatari S., Noda M., Miake K., Sugiyama M., Fujino T. (2012). Effects of plasmalogens on systemic lipopolysaccharide-induced glial activation and β-amyloid accumulation in adult mice. Ann. N. Y. Acad. Sci..

[28] Kantarci K., Senjem M.L., Lowe V.J., Wiste H.J., Weigand S.D., Kemp B.J., Frank A.R., Shiung M.M., Boeve B.F., Knopman D.S. (2010). Effects of age on the glucose metabolic changes in mild cognitive impairment. AJNR. Am. J. Neuroradiol..

[29] Herholz K., Salmon E., Perani D., Baron J.C., Holthoff V., Frolich L., Schonknecht P., Ito K., Mielke R., Kalbe E. (2002). Discrimination between Alzheimer dementia and controls by automated analysis of multicenter FDG PET. NeuroImage.

[30] Nieman B.J., de Guzman A.E., Gazdzinski L.M., Lerch J.P., Chakravarty M.M., Pipitone J., Strother D., Fryer C., Bouffet E., Laughlin S. (2015). White and gray matter abnormalities after cranial radiation in children and mice. Int. J. Radiat. Oncol. Biol. Phys..

[31] Scheef L., Spottke A., Daerr M., Joe A., Striepens N., Kolsch H., Popp J., Daamen M., Gorris D., Heneka M.T. (2012). Glucose metabolism, gray matter structure, and memory decline in subjective memory impairment. Neurology.

[32] Ashraf A., Fan Z., Brooks D.J., Edison P. (2015). Cortical hypermetabolism in mci subjects: A compensatory mechanism?. Eur. J. Nucl. Med. Mol. Imaging.

[33] Dubois A., Herard A.S., Delatour B., Hantraye P., Bonvento G., Dhenain M., Delzescaux T. (2010). Detection by voxel-wise statistical analysis of significant changes in regional cerebral glucose uptake in an APP/PS1 transgenic mouse model of Alzheimer’s disease. NeuroImage.

[34] Maier F.C., Wehrl H.F., Schmid A.M., Mannheim J.G., Wiehr S., Lerdkrai C., Calaminus C., Stahlschmidt A., Ye L., Burnet M. (2014). Longitudinal PET-MRI reveals β-amyloid deposition and RCBF dynamics and connects vascular amyloidosis to quantitative loss of perfusion. Nat. Med..

[35] Herholz K., Westwood S., Haense C., Dunn G. (2011). Evaluation of a calibrated 18F-FDG PET score as a biomarker for progression in Alzheimer disease and mild cognitive impairment. J. Nucl. Med..

[36] Doot R.K., Dunnwald L.K., Schubert E.K., Muzi M., Peterson L.M., Kinahan P.E., Kurland B.F., Mankoff D.A. (2007). Dynamic and static approaches to quantifying 18F-FDG uptake for measuring cancer response to therapy, including the effect of granulocyte CSF. J. Nucl. Med..

[37] Wang H., Liu J., Zong Y., Xu Y., Deng W., Zhu H., Liu Y., Ma C., Huang L., Zhang L. (2010). MIR-106B aberrantly expressed in a double transgenic mouse model for Alzheimer’s disease targets TGF-β type II receptor. Brain Res..

[38] Zhang L., Liu C., Wu J., Tao J.J., Sui X.L., Yao Z.G., Xu Y.F., Huang L., Zhu H., Sheng S.L. (2014). Tubastatin A/ACY-1215 improves cognition in Alzheimer’s disease transgenic mice. J. Alzheimer’s Dis..

[39] Zhang W., Gu G.J., Shen X., Zhang Q., Wang G.M., Wang P.J. (2015). Neural stem cell transplantation enhances mitochondrial biogenesis in a transgenic mouse model of Alzheimer’s disease-like pathology. Neurobiol. Aging.

[40] Shin J., Tsui W., Li Y., Lee S.Y., Kim S.J., Cho S.J., Kim Y.B., Leon M.J.D. (2011). Resting-state glucose metabolism level is associated with the regional pattern of amyloid pathology in Alzheimer’s disease. Int. J. Alzheimers Dis..

